# Protective effect of intranasal peste des petits ruminants virus and bacterin vaccinations: Clinical, hematological, serological, and serum oxidative stress changes in challenged goats

**DOI:** 10.14202/vetworld.2019.945-950

**Published:** 2019-07-03

**Authors:** Theophilus Aghogho Jarikre, Jeremiah Olalekan Taiwo, Benjamin Obukowho Emikpe, Stephen Owarioro Akpavie

**Affiliations:** 1 Department of Veterinary Pathology, Faculty of Veterinary Medicine, University of Ibadan, Nigeria; 2 Department of Veterinary Medicine, Faculty of Veterinary Medicine, University of Ibadan, Nigeria

**Keywords:** bacterines, goats, mucosal immunity, peste des petits ruminants virus

## Abstract

**Background and Aim:**

The current vaccination for peste des petits ruminants virus (PPRV) is stalled by myriad challenges and continuous endemicity of pneumonia due to fulminant bacterial complication in goats. The present study evaluated the protective effect of intranasal PPRV linage 1 and bacterine vaccinations.

**Materials and Methods:**

Twelve West African Dwarf (WAD) goats aged 6 months were randomly grouped and vaccinated within 2 weeks using a combination of PPRV lineage 1 vaccine (Nig/75), and bacterin from *Mannheimia haemolytica* (Mh) or *Pasteurella multocida* intranasally. The goats were observed for 3 weeks post-vaccination before comingled with a known infected WAD goat with apparent clinical signs of peste des petits ruminants and further observed clinically for 5 weeks post-infection (PI). Blood samples were taken for hematology while sera were assayed for antioxidants (glutathione peroxidase, glutathione transferase, and superoxide dismutase) activities and pro-oxidants (malondialdehyde content, reduced glutathione, hydrogen peroxide generation_,_ and myeloperoxidase) using spectrophotometric methods. Data were subjected to parametric statistics at α=0.05 using GraphPad Prism version 21.

**Results:**

Clinically, there were pyrexia, oculonasal discharge, diarrhea, anemia, leukopenia, and increased pro-oxidants in the unvaccinated goats, while moderate neutrophilia and leukocytosis were observed in PPRV and bacterin vaccinated goats. Two unvaccinated goats were weak and euthanized at 13 and 28 days PI. The goats vaccinated with PPRV and Mh showed better response clinically and biochemically.

**Conclusion:**

The mucosal vaccination of goats with PPRV vaccine and bacterine will protect against exposure and culminate in the development of protective mucosal, humoral, and cell-mediated immune responses. This vaccination strategy will provide framework needed in the prevention and control of endemic caprine pneumonia in Nigeria.

## Introduction

Respiratory diseases are induced by infectious agents, managemental conditions, and/or commingling among animals [1,2]. Our preliminary studies on goats from different regions of Nigeria have shown the dynamics, pattern and type, and risk factors of pneumonia in goats [2,3]. We also showed that vaccination through the intranasal route induced potent mucosal immunity in goat against peste des petits ruminants virus (PPRV) [4,5]. None the less, due to the complex nature of caprine pneumonia, it has defied all the attempts at control using only available vaccine hitherto. This probably may be due to the bacteria complications of primary viral infection [6] or presence of multiple pathogens [7] in caprine pneumonia. In Nigeria, the current vaccination for PPRV is stalled by myriad challenges, leading to continuous endemicity in goats.

Vaccination and immunity are hallmark of historical success of medicine against infectious diseases. More so, harnessing mucosal immune response is an important defense against invading pathogens [8]. Viral pathogens including PPRV, parainfluenza-3, and respiratory syncytial viruses in caprine pneumonia have been reported in our environment, while the role of bacteria including *Mannheimia haemolytica* (Mh) and *Pasteurella multocida* (Pm) was also investigated in some of our previous studies [6,9]. However, PPRV and Mh have been responsible for higher percentages of the pneumonic lesions in the goats. Thus, enhancing the mucosal immune response of goats to these organisms will considerably control losses from caprine pneumonia. It was observed that goats vaccinated intranasally against PPRV exhibited better clinical response [5] while that against Mh was not protective in the goats when clinically challenged with PPRV [10]. Thus, there is a need to vaccinate goats with both vaccines through the intranasal route and then challenge in an experimental model, to mimic field or natural conditions. This is to proffer different strategies and framework needed in the prevention and control of the endemic caprine pneumonia in Africa at large.

The present study evaluated the protective effect of intranasal PPRV linage 1 and bacterine vaccinations.

## Materials and Methods

### Ethical approval

The study was approved by the University of Ibadan Animal Care Use and Research Ethics Committee with the number UI-ACUREC/17/0060. The guidelines on the use of animals were duly followed and the animals were handled humanely to avoid pain.

### Experimental animals

Twelve West African Dwarf (WAD) goats 6 months old as described by Lasisi *et al*. [11] were acquired from a recognized breeding farm. They weighed between 4.5 and 6 kg each. The goats were housed in fly-proof pen at the experimental animal house of the Faculty of Veterinary Medicine. On arrival, they were acclimatized for 14 days and monitored clinically for changes in temperature, respiratory rate, body condition, etc. Blood, sera, and fecal samples were taken for screening using standard techniques for blood parasites (cytology), PPRV antibody enzyme-linked immune sorbent assay (ELISA), fecal protozoa, and helminths (floatation), respectively.

The animals were provided fresh water daily and maintained on ration of bean husks, maize bran, and forages (*Cynodon plectostachyus*) during acclimatization and period of experiment. They were further conditioned for 4 weeks before the start of experiment.

### Experimental design

The study followed a randomized complete block design, whereby the goats were randomly sorted into three groups comprising four goats each tagged on the neck for easy identification and then vaccinated within 2 weeks using PPRV lineage 1 vaccine Nig/75, a lyophilized vaccine sourced from National Veterinary Research Institute, Vom, Nigeria, and bacterines of Mh and Pm produced locally in our laboratory [12].

The animals were vaccinated as indicated in each group below.

- PPRV vaccine + Mh bacterin
- PPRV vaccine + Pm bacterin
- Control (not vaccinated)

About 1 ml of PPRV vaccine and 1 ml of the bacterin were sprayed into the nostrils of each goat. The animals were observed for 3-week post-vaccination.

### Experimental challenge

All the vaccinated animals were housed together under the same roof and a known confirmed infected WAD goat with apparent clinical signs of peste des petits ruminants (PPR) was introduced into the pen mimicking field infection [13,14]. All the animals were allowed to comingle and clinically observed daily for another 6-week post-infection (PI).

### Clinical examination

The respiratory rate was taken for each goat through the inhalation or expansion of the thoracic cavity for 60 s while the qualities of the respirations were noted. The body temperature was taken and recorded from the rectum using a sensitive thermometer. The general body condition was evaluated and scored systemically as described by Battaglia [15] on a scale of 1-3 (amount of fat and muscle at key anatomical points); 1= very thin (poor), 2= thin (fair), and 3= normal (good).

A 4 ml of blood was collected weekly from each goat by jugular venipuncture using a clean sterile disposable syringe and needle into heparinized and plain tubes for hematological, biochemical, and serological analysis.

### Hematological procedure

The hematological profiles were carried out manually as outlined by Jain [16]. Thin blood smears were made on clean glass slides, fixed in absolute methanol, and stained with Giemsa. The slides were viewed for blood parasites. Packed cell volume (PCV) was done using the Hawksley microhematocrit centrifuge set at 10,000 r.p.m for 5 min. PCV capillary tubes were read using the graphic reader. Plasma protein concentration was determined using the Goldberg refractometer (TS Meter, American Optical Scientific Instrument Division Buffalo, N.Y., U.S.A). Total red blood and white blood cell counts were determined using the improved Hawksley hemocytometer.

### Serum oxidative stress

Sera were assayed for antioxidants (glutathione peroxidase [GPx], glutathione transferase, and superoxide dismutase [SOD]) activities and pro-oxidants (malondialdehyde [MDA] content, hydrogen peroxide [H_2_O_2_] generation, reduced glutathione [GSH], and myeloperoxidase [MPO]) using spectrophotometric methods as previously described [9].

### ELISA

The PPR complement-ELISA kit designed to detect antibodies directed against the nucleoprotein of PPRV, developed by Food and Agriculture Organization reference laboratory (CIRAD-EMVT, Montpellier, France) was used to measure the PPRV antibody. The test was performed according to the instructions in the manufacturer’s manual.

### Statistical analysis

Experimental data were subjected to descriptive statistics and analyzed appropriately with parametric statistics at α=0.05 using GraphPad Prism version 21.

## Results

### Clinical observations

**Figure-1:**
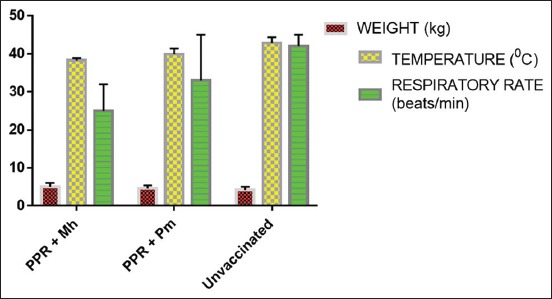
Clinical changes observed in goats intranasally vaccinated with peste des petits ruminants virus and *Mannheimia haemolytica* challenged goats.

Group A (PPRV + Mh vaccinated): There was no remarkable change in the weight of the goats (Figure-1). The goats showed slight pyrexia in the 1^st^ week post-vaccination, but which normalized 4 days later. There was a mild serous ocular discharge and the body condition was good.

Group B (PPRV + Pm vaccinated): The animals did not show signs of weakness until around the 11^th^ day post-infection (PI). There was moderate pyrexia, serous to purulent ocular discharge. One of the goats had a fair body condition score due to roughness of hair coat and dehydration.

Group C (Unvaccinated/control): There was pyrexia which peaked 4 days post-exposure. The goats were dehydrated due to severe diarrhea observed 1 week post-exposure. There were mucopurulent ocular discharge, pallor of mucous membranes, crusting and congestion of the conjunctiva, anorexia, dyspnea, and erosion on the dental pad. Two goats were very weak at the 13^th^ and 28^th^ days PI and were euthanized.

### Hematological observations

Group A (PPRV + Mh vaccinated): The hematocrit, hemoglobin concentration, and red cell counts were within reference range before and after vaccination, and 6 weeks PI. The leukocytes and platelets were also within reference range except the slight leukocytosis observed 6 weeks PI (α>0.05).

Group B (PPRV + Pm vaccinated): The erythrogram, leukogram, and platelet counts were also within range (Table-1).

**Table 1:** Hematological and serum protein changes in intranasal vaccinated peste des petits ruminants virus and *Mannheimia haemolytica* challenged goats.

Group	PCV* %	WBC×10^3^/µL	PLT×10^5^/µL	LYM×10^3^/µL	NEUT×10^3^/µL	NL*	MCV* fl	TP* g/dl	ALB* g/dl	IG* g/dl
Pre-vaccination										
A	32.3±2.3^a^	7.0±1.4^a^	1.4±0.3	5.3±1.2	1.7±0.2	0.4±0.0	27.9±0.9	8.4±0.1	3.5±0.1	4.9±0.1
B	33.0±2.7^a^	7.6±0.9^a^	1.4±0.2	5.4±0.6	2.2±2.4	0.4±0.0	29.1±1.7	7.9±0.3	3.1±0.3	4.8±0.0
C	28.3±3.2^a^	6.5±0.5^a^	1.4±0.3	5.4±0.3	1.1±0.3	0.4±0.1	26.4±2.5	8.2±0.3	3.5±0.3	4.7±0.2
Post-vaccination										
A	29.0±1.7^a^	13.3±4.6^a^	2.5±0.3	7.9±2.2	4.7±2.1	0.5±0.1	27.5±1.1	8.4±0.1	3.5±0.1	4.9±0.1
B	33.7±1.8^a^	11.4±3.9^a^	2.8±0.1	7.7±2.4	3.2±1.3	0.4±0.1	27.5±0.4	7.9±0.3	3.1±0.3	4.8±0.0
C	33.3±0.7^a^	7.6±2.2^b^	3.4±0.4	5.8±1.5	1.8±0.8	0.4±0.0	27.8±0.4	8.2±0.3	3.5±0.3	4.7±0.2
Post-infection										
A	27.0±1.5^a^	14.4±2.1^a^	2.0±0.5	6.6±0.5	6.8±1.6	1.0±0.2	20.1±1.3	6.5±0.3	2.6±0.1	3.9±0.2
B	17.7±0.9^b^	11.2±1.3^a^	1.7±0.3	5.6±0.9	5.0±0.2	0.9±0.1	18.7±2.1	7.3±0.4	3.2±0.4	4.1±0.0
C	11.7±6.0^c^	4.8±3.9^b^	1.1±0.6	2.1±2.2	2.7±1.6	0.5±0.3	16.0±8.2	4.7±2.4	1.9±1.0	2.8±1.4

PCV=Packed cell volume, HB=Hemoglobin concentration, RBC=Red blood cell, WBC=White blood cell, PLT=Platelets, LYM=Lymphocyte, NEUT=Neutrophil, NL=Neutrophil-lymphocyte ratio, MCV=Mean corpuscular volume, TP=Total protein, ALB=Albumin, IG=Immunoglobulin. Values with different superscript are significant

Group C (Unvaccinated/control): There were marked anemia, moderate leukopenia, thrombocytopenia, lymphocytopenia, neutrophilia, and marked hypoproteinemia few days PI in the goats (α<0.05).

### Serum protein and oxidative stress parameter changes

Group A (PPRV + Mh vaccinated): The plasma protein, albumin, and immunoglobulin values were within range and not different in the goats. The SOD, GSH, and GPx activities decreased slightly, while MPO content, MDA, and H_2_O_2_ generation increased (α>0.05).

Group B (PPRV + Pm vaccinated): The plasma protein, albumin, and immunoglobulin values were within range (Table-2). The SOD, GSH, and GPx activities decreased slightly, while MPO content, MDA, and H_2_O_2_ generation increased moderately (α<0.05).

**Table 2:** Serum oxidative stress parameter changes in intranasal vaccinated peste des petits ruminants virus and *Mannheimia haemolytica* challenged goats.

Group	SOD unit/mg p	GPx unit/mg p	GSH µg/mL	MPO µmol/min	MDA µmol/mL	H_2_O_2_ µmol/mg p
Pre-vaccination						
A	11.0±2.6^a^	16.0±4.5^a^	3.6±0.3^a^	1.1±0.5^a^	1.0±0.1^a^	1.5±0.3^a^
B	11.5±3.0^a^	16.4±5.5^a^	3.4±0.6^a^	1.8±1.0^b^	1.2±0.1^a^	1.0±0.5^b^
C	9.9±2.5^a^	15.8±4.8^a^	3.9±1.5^a^	2.8±1.0^c^	1.0±0.1^b^	1.5±0.7^b^
Post-vaccination						
A	10.0±2.5^a^	15.8±4.2^a^	8.8±2.1^a^	1.1±0.5^a^	1.0±0.1^a^	1.5±0.3^a^
B	11.3±3.1^a^	18.0±5.6^a^	9.1±2.7^a^	1.8±1.0^b^	1.2±0.1^a^	1.0±0.5^b^
C	9.9±2.8^a^	17.2±5.8^a^	6.0±3.1^b^	2.8±1.0^c^	1.0±0.1^b^	1.5±0.7^b^
Post-infection						
A	9.8±1.8^a^	16.4±2.3^a^	9.0±2.5^a^	2.2±1.5^a^	1.0±0.1^a^	3.2±2.2^a^
B	6.4±0.8^a^	12.6±1.9^a^	9.9±3.0^a^	14.8±2.9^b^	1.8±0.1^a^	8.0±2.5^b^
C	5.4±0.9^a^	9.4±2.2^a^	13.9±3.0^b^	24.5±3.0^c^	2.1±0.1^b^	9.5±2.9^b^

SOD=Superoxide dismutase, GPx=Glutathione peroxidase, GSH=Reduced glutathione, MPO=Myeloperoxidase, MDA=Malondialdehyde, H_2_O_2_=Hydrogen peroxide. Values with different superscript are significant

Group C (Unvaccinated/control): There were marked hypoproteinemia, hypoalbuminemia, and hypoglobulinemia (α<0.05). There were general reductions in SOD and GPx activities and increased MPO content, MDA, GSH, and H_2_O_2_ generation (α<0.05).

### Serology

**Figure-2:**
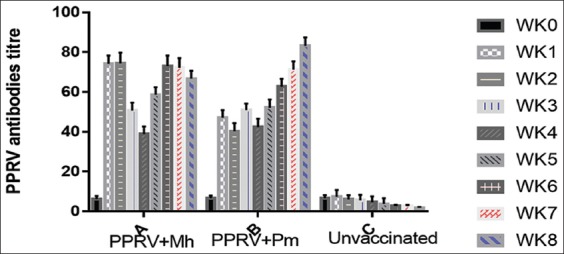
Percentage inhibition titer of peste des petits ruminants virus antibodies from sera of goats intranasally vaccinated with peste des petits ruminants virus and Mh challenged goats using complement-enzyme linked immunosorbent assay.

The percentage inhibition titer of PPRV antibodies in the goats is shown in Figure-2. The highest titer was in the goats vaccinated with PPRV + Mh and the least titer in unvaccinated goats (α<0.05).

## Discussion

This present study evaluated the protective effect of intranasal PPRV and *bacterin* vaccination in challenged goats. Viral complicated bacterial pneumonia is very fulminating in small ruminants and causes losses of income to farmers [17]. The intranasal combined PPRV and Mh vaccines elicited better protection against caprine pneumonia in the WAD goats.

The clinical changes observed in the infected goats were similar to earlier findings in both experimental and natural infections of PPR in goats [6,18]. All the unvaccinated goats showed severe signs of respiratory distress due to the pulmonary consolidation and blockage of the nasal passages and airways. The anemia, leukopenia, and hemoconcentration observed in goats with clinical pneumonia were similar to that reported by Obi *et al*. [19], Olaleye [20], and Ezeasor *et al*. [5]. The dehydration in the infected animals probably contributed significantly to the weakness of the animals.

The intranasal vaccination with combined PPRV vaccine and Mh bacterine protected the goats better than other schedules in the groups of comingled goats. This may be due to the strong mucosal response elicited by the PPRV and Mh vaccines or their synergistic activity on the induction of immune response antigenically.

The harnessing of the mucosal route has been demonstrated as a potent initiator of protective immune response from our findings. This was also similar to reports on pneumococcal vaccination of mice through the intranasal route which boosted the mucosal immunity against *Streptococcus pneumoniae* [21]. Ezeasor *et al*. [5] also reported better response from intranasal PPRV vaccination; however, Mh bacterine immunization alone was not protective to goats in our environments [10,22], it is now clear that a combination of PPRV vaccine and the bacterine provided better protection to caprine pneumonia.

The reason for comingling the goats was justified by the fact that outbreaks of PPR occur when new stock is introduced into a naïve flock [13]. More so, comingling resulted in fulminant PPR because all the goats will be exposed to the virus in excretions and secretions from the sick goat.

From this study, PPRV caused severe necrotizing and hemorrhagic enteritis, evident clinically as diarrhea and anemia which is similar to reports of Olaleye [20] and Sahinduran *et al*. [23]. Furthermore, the leukopenia, monocytopenia, and lymphopenia arise because the virus replicates in lymphoid tissues using signaling lymphocyte adhesion molecules. Our clinical and hematological findings were similar to reports of Aikhuomobhogbe and Orheruata [24] and Sahinduran *et al*. [23]. Yarim *et al*. [25] also reported the significant effect of PPRV-induced thrombocytopenia in young goats.

Enhanced mucosal response can be assessed by the expression of secretory immunoglobulin A antibody [8,26]. This may have been reflected in the hyperglobulinemia observed in the vaccinated animals, which showed better immune response. However, the expression comprises antibody isotypes, cytokine, complement, and adaptive immune cells.

## Conclusion

In conclusion, intranasal combined PPR and Mh vaccines elicited better protection against pneumonia in goats. This vaccinal strategy will provide framework needed in the prevention and control of endemic caprine pneumonia in Nigeria.

## Authors’ Contributions

The results reported herewith are part of TAJ’s doctoral thesis. BOE and SOA designed the work. TAJ and JOT did the experiments and collected the results. TAJ and BOE compiled the results and drafted the manuscript. All authors read and approved the final manuscript.
